# Plasma Carotenoids and Polyphenols and Their Association with MetS: The Need for Nutritional Interventions

**DOI:** 10.3390/antiox12071336

**Published:** 2023-06-24

**Authors:** Agnieszka Białkowska, Magdalena Górnicka, Monika A. Zielinska-Pukos, Ewelina Hallmann, Jadwiga Hamulka

**Affiliations:** 1 Department of Human Nutrition, Institute of Human Nutrition Sciences, Warsaw University of Life Sciences (SGGW-WULS), 02-787 Warsaw, Poland; agnieszka_bialkowska@sggw.edu.pl (A.B.); magdalena_gornicka@sggw.edu.pl (M.G.); monika_zielinska_pukos@sggw.edu.pl (M.A.Z.-P.); 2 Department of Functional and Organic Food, Institute of Human Nutrition Sciences, Warsaw University of Life Sciences (SGGW-WULS), 02-787 Warsaw, Poland; ewelina_hallmann@sggw.edu.pl

**Keywords:** metabolic disorders, MetS severity, carotenoids, polyphenols, lipids profile, glucose, inflammation, adults

## Abstract

Metabolic syndrome (MetS) is characterized by increased pro-oxidative stress and a chronic inflammation state and their consequent alterations. Several studies have highlighted the protective effect of carotenoids and polyphenols in MetS patients. This study aimed to evaluate the plasma level of selected carotenoids and polyphenols and to determine their relationship with MetS severity, MetS components, and inflammatory markers in Polish adults with metabolic disorders. It was designed as a cross-sectional study. The final study group comprised 275 adults, including 158 women and 117 men. Data were collected on the frequency of consumption of selected food groups. Anthropometric measurements and blood samples were taken to determine the concentration of carotenoids, polyphenols, and indicators (parameters) of metabolic disorders. Plasma concentrations of selected carotenoids and polyphenols were low in adults with MetS. The highest concentrations of carotenoids and polyphenols in the blood were observed for lutein and phenolic acids (including gallic and p-coumaric acids). Nevertheless, a correlation was found between the individual bioactive compounds and MetS components. In terms of the lipid profile, our study showed that the plasma of the selected carotenoids and polyphenols positively correlated with HDL cholesterol (zeaxanthin; total carotenoids), LDL cholesterol (chlorogenic acid), triglycerides (lycopene), and the total cholesterol (kaempferol). We found that the level of CRP as a marker of inflammation negatively correlated with the concentration of zeaxanthin. In our study group, no relationship was found between the dietary antioxidant intensity and the variables studied, which may be attributed to the low frequency of consumption of the sources of bioactive compounds, such as carotenoids and polyphenols, but also to the metabolic disorders. Further research is needed to determine whether these associations are causally related to the metabolic syndrome or are a result of the pathologies of the syndrome or improper diet with a low intake of vegetables and fruit.

## 1. Introduction

Potentially beneficial anti-inflammatory and antioxidant effects are exhibited by naturally occurring plant-derived bioactive components such as carotenoids and polyphenols [1,2,3]. Carotenoids are a group of naturally occurring lipophilic pigments synthesized by plants, algae, and photosynthetic bacteria but not humans, who must ingest them in food or via supplementation. They are mainly found in vegetables and fruits but are also available in other sources such as eggs, beverages, fats, and oils. Of the more than 40 carotenoids in the human diet, only β-carotene, α-carotene, β-cryptoxanthin, lycopene, lutein, and zeaxanthin are detectable in human serum. Carotenoids have many important functions such as antioxidant, antibacterial, immunological, and anti-inflammatory activity and have beneficial effects in the treatment of infections [4,5]. Meta-analysis results [6] showed that serum levels of carotenoids, especially α- and β-carotene and retinyl esters, were inversely related to the MetS syndrome. Moreover, Harari et al. [7] found that insulin resistance in obesity was inversely associated with serum and adipose tissue carotenoid concentrations in adults. However, further research is needed to establish the relationship and suboptimal levels for the dietary prevention of metabolic disorders.

Polyphenols have also been shown to have antioxidant, anti-inflammatory, and antithrombotic properties. They can improve vascular endothelial functions, insulin secretion, and can lower blood pressure, which is highly beneficial in treating cardiometabolic disorders [8,9,10]. Polyphenols are a group of compounds divided into five main classes: flavonoids, phenolic acids, stilbenes, lignans, and others, found in foods and beverages commonly consumed by humans [11,12,13]. Several experimental studies provided suggestive evidence that an increased intake of flavonoid-rich foods may reduce the risk of cardiometabolic diseases, although the authors also note the challenge of the high heterogeneity and diversity of phenolic subclasses and food sources [8]. 

Obesity, such as dyslipidemia and hyperinsulinemia, is associated with oxidative stress [1,3,12], characterized by the pro-oxidant–antioxidant balance (PAB) shifting toward the pro-oxidant predominance, resulting in molecular damage and further necessitating the use of antioxidants such as carotenoids and polyphenols to inactivate free radicals [6,14,15,16,17]. Increased oxidative stress and associated low-grade chronic inflammation caused by complex interactions between adipokines and adipocytokines are characteristic of the obesity state found in metabolic syndrome (MetS) [17,18,19]. 

The currently observed increase in the MetS trend is consistently associated with an increased risk of type 2 diabetes (T2D), cardiovascular diseases (CVD), and non-alcoholic fatty liver disease (NAFLD). Thus, MetS is a major global public health problem, increasing mortality, disabilities, and healthcare costs [20,21,22]. Therefore, modifiable lifestyle factors (reduced caloric intake, high intake of antioxidant compounds, increased physical activity) and relationships between bioactive compounds and MetS components that may reduce the burden of MetS have become the subject of many studies in recent years [6,23,24].

Due to the limited evidence on plasma carotenoids’ and polyphenols’ concentration in Polish adults with MetS and the fact that MetS is widespread in our country, this study was undertaken. This study aimed (1) to evaluate the plasma level of selected carotenoids and polyphenols and (2) to determine their relationship with MetS severity, MetS components and inflammatory markers in Polish adults with metabolic disorders.

## 2. Materials and Methods

### 2.1. Ethics Approval

This study was conducted according to the guidelines outlined in the Declaration of Helsinki and the Ethics Committee of the Faculty of Human Nutrition and Consumer Science at the Warsaw University of Life Sciences, Poland, on 11 April 2017 (Resolution No. 04p/2017). Before enrolling, all participants provided written informed consent to participate in the study. Additionally, before commencing the interview and measurements, the participants were duly informed about the study’s objectives and potential risks.

### 2.2. Study Design and Participants

This study was designed as a cross-sectional study with convenience sampling. As part of the research project, this study encompassed a thorough evaluation of the following: (1) dietary intake, (2) an analysis of anthropometric measurements and body composition, (3) an assessment of metabolic syndrome indicators and inflammation, and (4) a measurement of the plasma levels of carotenoids and polyphenols in individuals with metabolic disorders. Simply put, the individuals involved in this study were selected from patients who received treatment at the Metabolic Diseases Outpatient Clinic in Warsaw, following their initial assessment by a general practitioner. The inclusion criteria were the following: Caucasian and with an age of over 18 years old; a diagnosis of metabolic diseases including obesity, diabetes, hypertension, or/and dyslipidemia (diagnosis of metabolic syndrome); and a signed informed consent from the participants. The exclusion criteria were the following: pregnant or breastfeeding (for women); people with acute chronic diseases (cancer, kidney failure, and diabetic ketoacidosis) and with large fluctuations in body weight in the last 6 months; and people taking medications that may have affected the results of this study and taking supplements with carotenoids and/or polyphenols. Detailed information on the sample recruitment and the methodology and procedures applied was published previously [25].

### 2.3. Data Collection

#### 2.3.1. Socio-Demographics and Lifestyle Data

Questionnaires were used and filled out by a well-trained researcher to limit ambiguity. The questionnaires included questions about age, sex, education, employment and socioeconomic status, medical history (self-reported illness history and current medications), lifestyle factors (diet, dietary supplements, physical activity, smoking status, and alcohol consumption), and a family history of chronic diseases.

#### 2.3.2. Dietary AntiOxi Index

A Dietary Habits and Nutrition Beliefs Questionnaire (KomPAN) [26,27] was used to assess the frequency of consumption of the selected food groups. The Dietary AntiOxi Index (AOI) was calculated based on the daily frequency intake of chosen groups of products widely known as sources of carotenoids and polyphenols. The participants were asked about the frequency of consumption of products such as vegetables, fruits, whole grains, legumes, and beverages such as tea, herbs, coffee, cacao, juices, compotes, nectars, and vine. Participants were asked how often they had eaten each type of food in the past three months. Seven categories could be chosen: 1—never or almost never; 2—less than once a week; 3—once a week; 4—2–4 times a week; 5—5–6 times a week; 6—times a day; and 7—several times a day. The obtained frequency answers were converted into daily frequencies and consisted of the following: never (0 times/day), 1–3 times a month (0.06 times/day), once a week (0.14 times/day), a few times a week (0.5 times/day), once a day (1.0 time/day), or a few times a day (2.0 times/day); detailed information in this scope was published previously [26]. The dietary score was created a priori by summing the consumption frequencies (times/day). Food group consumption levels were classified on a percentage scale with respect to the sum of the recommended value (max. 16.16–100%). The AOI was expressed in % points and categorized as follows: low (0–33.32% points), moderate (33.33–66.65% points), and high (66.66–100% points) antioxidant intensity of the diet.

#### 2.3.3. Anthropometry Measurements

A well-trained researcher collected anthropometric data from the participants using standardized techniques and calibrated equipment. Body weight (BW), height (H), waist (WC), and hip (HC) circumferences were measured. Body composition, including fat mass (FM) and fat-free mass (FFM), was assessed by a bioelectrical impedance technique using a multi-frequency (MF) eight-point Tanita Analyzer (Tanita BC-418 MA, Tanita Co., Tokyo, Japan). Measurements were taken under strictly standardized conditions, twice in light clothing and without shoes, and the averages were calculated. 

#### 2.3.4. Blood Pressure

Systolic and diastolic blood pressures (SBP and DBP, respectively) were measured in a sitting position after resting at least 10 min using a standardized sphygmomanometer following the best practice/was consistent with the National Institute for Health and Care Excellence (NICE) [28]. Two consecutive readings on the same arm were recorded, and their results were averaged for further analysis.

#### 2.3.5. Blood Sample

The day before sampling, participants were asked to fast for at least 12 h and not drink fruit juices. Blood samples were collected in the morning, between 7 a.m. and 9 a.m., by standard venipuncture procedures. The collected blood was centrifuged for 10 min at 5000 rpm at 4 °C and stored frozen (−80 °C) for further analysis.

#### 2.3.6. Biochemical Analysis

All analyses were performed in a certified laboratory using standard methods. Total cholesterol (CHOL) concentration was determined using the enzyme method with esterase and cholesterol oxidase. High-density lipoprotein (HDL-C) and triglyceride (TG) were determined through the colorimetric non-precipitation method. The LDL cholesterol blood level (LDL-C) was calculated using the Friedewald formula [29]. The fasting plasma glucose (FPG) concentration was measured using the enzyme method with hexokinase. C-reactive protein (CRP) concentration was determined using the immunoturbidimetric method.

According to the definition by The Centers for Disease Control and Prevention (CDC) and the American Heart Association (AHA), based on CRP concentrations, people were classified into 3 groups depending on the degree of risk of cardiovascular diseases and coronary incidents: <1.0 mg/L—low risk; 1.0 to 3.0 mg/L—moderate risk; >3.0 mg/L—high risk [30].

The concentration of carotenoids was determined using a high-performance liquid chromatography (HPLC) method adapted from Wu et al. [31]. Before analysis, the blood samples were saponified based on the modified methods [32,33]. The concentrations of the selected carotenoids (β-carotene, lycopene, and lutein + zeaxanthin) were determined using a HPLC system (Shimadzu, Kyoto, Japan)—two pumps LC-20AD, CMB-20A controller system, SIL-20AC autosampler, UV/IS SPD-20AV and SPD-M20A detectors, and CTD-20AC controller, using C18 Synergi Max-RP 80i columns (250 × 4.60 mm). The concentration of the carotenoids was assessed based on standard curves prepared with Sigma Aldrich and Merck standards and expressed in µmol/L. The results were obtained from chromatograms and calculated according to previously prepared standard curves. Isocratic phases composed of acetonitrile, methanol, and ethyl acetate were used for the analysis. The flow rate was 1 mL/min, and there was a wavelength of 445 nm for the xanthophylls and of 471 nm for the carotenes. 

The concentration of the polyphenols was analyzed by the high-performance liquid chromatography (HPLC) method adapted from Radtke [34]. The concentrations of the selected polyphenols were determined using a Shimadzu HPLC system (Shimadzu, Kyoto, Japan), described in detail previously, using C18 Synergi Fusion-RP 80i columns (250 × 4.60 mm). Individual phenolics were identified based on spectra identification and chromatogram pictures. The concentration of individual polyphenols was assessed based on standard curves prepared with Sigma Aldrich and Merck standards. The isocratic phases were composed of acetonitrile (55% and 10% with pH 3.0) and deionized water. The flow rate was 1 mL/min, and there was a wavelength of 240 nm for the phenolic acids and of 340 nm for the flavonoids.

#### 2.3.7. Diagnosis of Metabolic Syndrome and MetS Severity

The International Diabetes Federation (IDF) criteria [22] were used to diagnose MetS. Participants were categorized as MetS if they had metabolic abnormalities based on the following cut-off points: central obesity (≥94 cm in males and ≥80 cm in females; or BMI > 30 kg/m^2^, thus assuming central obesity). Categorization was also based on two of the following factors: fasting plasma glucose (Glu) ≥ 100 mg/dL; elevated triglycerides (≥150 mg/dL) or being treated for it; low HDL-C (<40 mg/dL in males and <50 mg/dL in females) or being treated for it; and elevated BP (systolic SBP ≥ 130 or diastolic DBP ≥ 85 mmHg) or treatment for hypertension.

Based on the individual values of MetS components and equations proposed by DeBoer and Gurka [35], we calculated continuous variables reflecting MetS severity:

Non-Hispanic White Males = −5.4473 + 0.0125 × waist circumference (cm) − 0.0251 × HDL (mg/dL) + 0.0047 × SBP (mmHg) + 0.8244 × ln(Tri) + 0.0106 × Glu (mg/dL);

Non-Hispanic White Females = −7.2536 + 0.0254 × waist circumference (cm) − 0.0120 × HDL (mg/dL) + 0.0075 × SBP (mmHg) + 0.5800 × ln(Tri) + 0.0203 × Glu (mg/dL).

### 2.4. Study Group Characteristics

Finally, the study group consisted of 275 adults, including 158 women (58%) and 117 men (42%), with an average age of 54 years (Table 1). Most of them (73%) had a secondary or higher education. Only 25% declared moderate or high physical activity. A similar percentage declared smoking during the study. The mean values of the AntiOxi Index in this group characterized diet as moderate antioxidant intensity. Taking into account the frequency of consumption of the selected product groups, it should be noted that in this study group, the highest daily frequency was found for tea, fruit, and coffee. Mean values of BMI, WC, WHtR, and FM (%) indicated adiposity, while biochemical markers confirmed metabolic disorders. Over half of the group had MetS components of 3. Values of MetS severity were 0.8 for women and 0.5 for men.

### 2.5. Statistical Analysis

Data were presented as a sample percentage (%) for categorical data or mean and standard deviation (SD) and median for continuous data. Before statistical analysis, we conducted the Shapiro–Wilk test to assess the normality of distribution and the Levene test to evaluate the homogeneity of variance. We divided the results according to MetS components (3–5), AntiOxi quartile, and the risk of the cardiovascular incident based on CRP results. The differences between groups were assessed by a Chi2 test for categorical variables, and ANOVA (for parametric distributions) or ANOVA Kruskal–Wallis tests (for nonparametric distributions) with a post-hoc analysis where necessary.

The multivariate linear regression models were calculated to evaluate the associations between plasma carotenoids and polyphenols and the individual MetS components (fasting glucose, HDL-C, triglycerides, WC, and systolic and diastolic blood pressure), LDL-C, cholesterol, CRP, and MetS severity. Models were adjusted for sex, age, fat mass (%), education, smoking status, and physical activity. Results of the analysis were expressed as β-coefficients with a 95% confidence interval, and for each model, the R^2^ value was calculated. For the polyphenols, we presented only significant models. Statistical significance was determined with a *p*-value < 0.05. The statistical analysis was performed using the STATISTICA software version 13.0 (StatSoft Inc., Tulsa, OK, USA).

## 3. Results

### 3.1. Effect of MetS and Inflammation on Plasma Bioactive Compounds

#### 3.1.1. Plasma Carotenoids and Number of MetS Components

Among the carotenoids, the highest plasma concentration was found for lutein (Table 2). Taking the number of MetS components into account, the level of the total carotenoids as well as lutein, zeaxanthin, and the sum of lutein/zeaxanthin were significantly lower in subjects with five MetS components. There were no significant differences between the number of MetS and the plasma level of β-carotene.

#### 3.1.2. Plasma Polyphenols and Number of MetS Components

The total plasma polyphenol concentration was 79.3 nmol/L, of which 77% were phenolic acids (Table 3). Among the phenolic acids in the plasma, p-coumaric acid, gallic acid, and caffeic acid predominated (32%, 26%, and 19%, respectively). The remainder were flavonols, of which quercetin was the predominant. There were no significant differences between the number of MetS and the plasma level of polyphenols.

**Figure 1:**
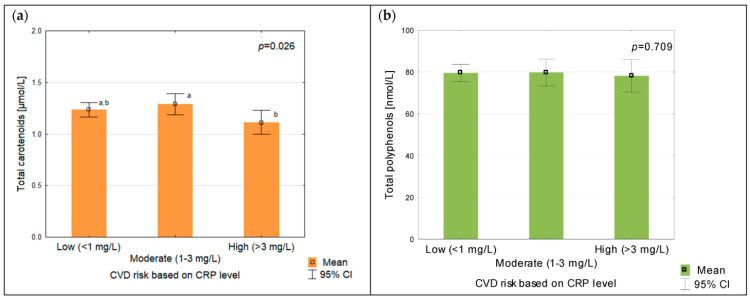
Total plasma carotenoids (**a**) and polyphenols (**b**) according to CVD risk based on CRP levels; different letters indicate that the samples are significantly different at *p* < 0.05.

Significantly lower concentrations of the total carotenoids were found in people with CRP levels indicating a high degree of CVD risk (Figure 1). For the polyphenols, no relationship was found.

### 3.2. Correlation between AntiOxi Index and Plasma Antioxidants and MetS Severity

Table 4 shows that only individuals with the highest values of the AntiOxi Index (Q4) had higher plasma lycopene concentrations. Moreover, weak positive partial correlations (r = 0.171; *p* = 0.008;) were found between the plasma lycopene concentration and between the total plasma carotenoids and the AntiOxi Index (r = 0.128; *p* = 0.047;).

### 3.3. Association between Plasma Bioactive Compounds and MetS Severity, Individual MetS Components and CRP

#### 3.3.1. Plasma Carotenoids

The analysis of the correlation between MetS severity and plasma carotenoids revealed a non-significant association, after adjusting for potential confounders (sex, age, fat mass, education, smoking status, physical activity, and AntiOxi Index) (Table 5). Correlations were only found for the individual components of MetS and CRP as a marker of inflammation. A significant negative association was found between fasting glucose and lutein (β: −0.126; CI: −0.249, −0.003), lutein/zeaxanthin (β: −0.134; CI: −0.258, −0.010), and the total carotenoids (β: −0.148; CI: −0.271, −0.025). There was a positive association between HDL-cholesterol and zeaxanthin (β: 0.157; CI: 0.042, 0.272), lutein/zeaxanthin (β: 0.125; CI: 0.008, 0.241), and the total carotenoids (β: 0.128; CI: 0.012, 0.243), as well as between TG and lycopene (β: 0.166; CI: 0.046, 0.286). WC was negatively associated with lutein (β: −0.079; CI: −0.152, −0.007) and lutein/zeaxanthin (β: −0.091; CI: −0.164, −0.017). The sum of these carotenoids was also associated with decreasing DBP (β: −0.122; CI: −0.243, −0.001). Plasma zeaxanthin and the total carotenoids were negatively associated with CRP (β: −0.153; CI: −0.276, −0.031; β: −0.123; CI: 0.243, −0.003).

#### 3.3.2. Polyphenols

After adjusting for potential confounders (sex, age, fat mass, education, smoking status, and physical activity), no significant association was found between the plasma polyphenols and MetS severity (Table 6). A significant negative association was found between p-coumaric acid and fasting glucose (β: −0.140; CI: −0.260, −0.021). There was a positive association between chlorogenic acid and LDL-cholesterol (β: 0.138; CI: 0.020, 0.256) and between kaempferol and the total cholesterol (β: 0.127; CI: 0.008, 0.247).

## 4. Discussion

The plasma concentration of the selected carotenoids and polyphenols and the frequency of consumption of their sources was low in adults with MetS. Among the carotenoids and polyphenols, the highest concentrations in the blood were observed for lutein and phenolic acids, respectively (including gallic and p-coumaric acids). However, a weak correlation has been discovered between individual bioactive compounds and MetS components. Regarding the lipid profile, our study showed that the plasma of the selected carotenoids and polyphenols was positively correlated with HDL cholesterol (zeaxanthin; the total carotenoids), LDL cholesterol (chlorogenic acid), triglycerides (lycopene), and the total cholesterol (kaempferol). It was found that the level of CRP as a marker of inflammation was negatively correlated with the concentration of zeaxanthin and the total carotenoids. Lower plasma carotenoids were associated with more MetS components and inflammation and consequently a higher CVD risk. 

The low levels of plasma carotenoids in our study group may be related to a low intake of their dietary sources. As pointed out by Toh et al. [36], plasma carotenoids are biomarkers of both vegetable, fruit, and carotenoid intake. However, when carotenoid biomarkers are used, it is worth noting that they may be susceptible to sociodemographic and other dietary factors, which should be considered in a more comprehensive assessment. The bioavailability and absorption of carotenoids are limited by factors such as their dietary amount and sources, food matrix and carotenoid location, food heating and processing, season and food composition, especially the intake of fat, dietary fiber, and protein and other compounds [37,38,39,40]. As found by Marhuenda-Muñoz et al. [41], a very high dietary fat intake lowered carotenoid concentrations in plasma. They indicated the need for further clinical trials with varying amounts of fat to determine the impact on plasma biomarkers related to fruit and vegetable consumption. The objective is to determine if regulating dietary fat intake, in terms of type and quantity, is necessary for optimal nutrition outcomes. Furthermore, carotenoids, like other fat-soluble vitamins, i.e., vitamins E and D, accumulate in the adipose tissue but are also stored in the liver, the eye macula, kidneys, lungs, ovaries, testes, and pineal gland; hence, their lower plasma levels are observed [14,41,42,43].

In our study, for individual carotenoids, such as lutein and/or zeaxanthin, as well as the total plasma carotenoids, an inverse association with fasting plasma glucose was found. This finding is consistent with other studies that have demonstrated the potential for carotenoids not only to prevent but also treat or alleviate diabetes and its subsequent complications. Dietary carotenoids and plasma β-carotene negatively correlated with fasting plasma glucose, insulin resistance, and HbA1c [7]. A similar relationship was found between serum carotenoids (lycopene, lutein, and β-carotene) and fasting serum insulin and the metabolic syndrome [44]. In addition, meta-analysis and systematic review results [6] showed that the total and individual carotenoids were inversely related to MetS. The strongest association was found for β-carotene, followed by α-carotene and β-cryptoxanthin. 

It has been proposed that, of the carotenoids, only β-carotene has optimum recommended concentrations to reduce the risk of ischemic heart disease, set at >0.4 μmol/L [45]. In our study group, the average concentration, regardless of MetS severity, was below the recommended level. We found that plasma β-carotene concentrations decreased with increasing MetS severity in adults with MetS. Similarly, the level of the total carotenoids, including lutein, zeaxanthin, and the sum of lutein/zeaxanthin, were significantly lower in subjects with five MetS components. These findings confirm the inadequate consumption of vegetables and fruits as sources of carotenoids in patients with higher MetS severity. 

High plasma concentrations of polyphenol metabolites may reflect the regular and frequent consumption of specific plant products. As examined in the EPIC cohort, the main food items that predicted concentrations of combined polyphenols included cereal-based products and sauces (i.e., soya sauce and tomato sauce), coffee, and tea [46]. In our study group, the daily frequency of intake of potential antioxidant food was low. Among the products considered, the highest daily frequency was for tea, coffee, and fruits, consumed on average once a day. In addition, the respondents declared the addition of sugar, milk, or cream to drinks, which must be considered in further research on bioavailability and diet therapy.

We found that among the polyphenols, phenolic acid as a p-coumaric acid and gallic acid were the most detectable in plasma, although no association with MetS severity was observed. Further analysis indicated that only three phenolic compounds were associated with MetS components: p-coumaric acid inversely correlated with the fasting glucose level, but chlorogenic acid was positively correlated with LDL and kaempferol with the total cholesterol. According to our findings, phenolic acids have potential benefits in managing diabetes, which is consistent with previous studies [20,47]. The antihyperglycemic effects of p-coumaric acid and gallic acid have been elucidated in an animal model. They were found to be involved in delaying intestinal glucose absorption, increasing β-cell activity, and promoting glucose uptake by peripheral tissues by enhancing insulin sensitivity [48].

Chlorogenic acid is one of the main phenolic acids and is present in coffee and various fruits and, as a nutraceutical, has been proven to be an important compound in the prevention and treatment of MetS due to its antioxidant, anti-inflammatory, hypolipidemic, antidiabetic, and antihypertensive properties [21,49]. Despite other research results, which found that high doses of chlorogenic acid can significantly reduce the total and LDL cholesterol levels and increase HDL cholesterol levels [50] by modulating the peroxisome proliferator-activated receptor (PPAR) γ responsible for the lipid metabolism [51], the finding in our study differs. It can be assumed that in our group, its positive correlation with LDL-cholesterol may be associated with coffee consumption. As noted by Schoeneck and Iggman [52], LDL cholesterol can increase with unfiltered coffee, as well as sugar, which is often added to coffee as well.

Similarly, the observed relationship between kaempferol and cholesterol is not confirmed in the results of studies by other authors. Animal studies have indicated antioxidant and anti-inflammatory effects and cardioprotective and antihypertensive benefits for kaempferol, but still little is known about its absorption from food and its potential cardioprotective benefits in humans [53]. However, as Millar et al. [54] point out, the doses and concentrations of flavonoids used in preclinical studies may be difficult to obtain solely from the human diet. Moreover, in humans, the bioactivity of these compounds depends on their absorption and metabolism. Therefore, the consumption of flavonoids as nutraceuticals requires more clinical trials to demonstrate their clinical value in metabolic syndrome treatments. While flavonoid compounds have demonstrated beneficial impacts on insulin resistance, plasma glucose reduction, the enhancement of the lipids profile, and cardiovascular protection in animal models and in vitro experiments, further clinical trials and in vivo experiments are required to confirm their efficacy [55]. 

As Koudoufio et al. [56] highlighted in their critical review, polyphenols, especially those with a high molecular weight, exhibit poor bioavailability, water solubility, permeability, and instability. In addition, the intestinal microflora is of great importance in the biotransformation of polyphenols into active metabolites. The metabolites present in the blood circulation result from digestive and hepatic activity; therefore, individual gut microbiota and metabolism should be considered when interpreting human study data. This microbial metabolization of dietary flavonoids has been considered a pivotal process for bioaccessibility and bioavailability. Moreover, polyphenols or their metabolites modulate the taxonomic composition of gut microbiota (“a two-way reciprocal relationship concept”). In this context, the role of flavonoid intake in the gut microbiota modulation is a key approach for improving health and preventing chronic diseases [57]. Similarly, the bioavailability of carotenoids in nature is limited, and there is insufficient knowledge on their pathway through the gastrointestinal tract regarding inflammation and related digestive processes. Additionally, the role of intestinal microbiota and their metabolites in the metabolism and absorption of carotenoids remains unclear based on the research conducted [37,38,58].

Dietary intervention studies in people with MetS indicate the potential therapeutic potential of carotenoids and polyphenols [59,60,61,62,63,64]; however, the results of clinical trials have been inconsistent [65,66,67]. Consuming a balanced and bioactive-rich diet low in carbohydrates and saturated fat is critical to managing metabolic syndrome. These compounds are key for the design and development of more effective compounds to support human health [21,24]. In general, our study revealed low plasma levels of individual carotenoids and polyphenols, possibly related to several aspects. Firstly, we included people who have been diagnosed with a metabolic syndrome (at least three components of MetS). Secondly, our respondents consumed small amounts of vegetables and fruits, which are primary sources of bioactive compounds in the diet. To demonstrate the correlation between typical consumption and biochemical parameters, we opted to exclude individuals who were supplementing their diet with carotenoids and polyphenols, in accordance with our exclusion criteria outlined in Section 2.2. Thirdly, the levels of the tested carotenoids in the blood are influenced by many factors that limit or increase their bioavailability, including the composition of the diet, e.g., the consumption of fat, dietary fiber, or the method of food preparation. As highlighted by Grosso et al. [23], food preferences specific to a given country or region may lead to discrepancies in research results. 

Taken together, our study results and current evidence suggest that carotenoids’ and polyphenols’ intake and their plasma levels and metabolites have the potential to lower inflammation and MetS-related markers [46,68,69]; however, no carotenoids/polyphenols or carotenoids-/polyphenols-rich food can influence all MetS features [70]. A varied diet based on foods rich in polyphenols and carotenoids, mainly in the form of health-promoting nutritional models (the Mediterranean diet, DASH, and the Okinawa diet) may be beneficial at the onset and progression of MetS and in reducing the associated risk of developing diabetes or cardiovascular diseases [71]. Therefore, the recommendation of diets, including the Mediterranean diet and the Okinawa diet as models of nutrition (dietary patterns) based on green and yellow vegetables, root vegetables, soybean-based foods, and other plants, supplemented by regular seafood consumption and the consumption of smaller amounts of lean meats, as well spices and tea, is crucial and can contribute to improving biochemical indices and reducing the risk of CVD and MetS [71,72].

As emphasized by Singh et al. [73], the Mediterranean diet, the DASH diet, and the Japanese diet can significantly reduce incidences of cardiovascular diseases and diabetes and can be used both in the prevention and treatment of metabolic diseases. However, as the best solution, they indicate the Indo-Mediterranean diet, similar to the traditional Japanese diet (with a higher content of flavonoids: 1800 vs. 1500 mg/day, respectively). The Indo-Mediterranean diet was shown to be more effective than also a conventional prudent diet due to its greater food variety, lower glycaemic index, and lack of unhealthy animal-based foods. The Indo-Mediterranean diet contains millets, porridge, and beans, as well as spices such as turmeric, cumin, fenugreek, and coriander, which may have better anti-inflammatory and cardioprotective effects. These foods are rich sources of nutrients, flavonoids, calcium, and iron, as well as proteins, which are useful in the prevention of under- and overnutrition and related diseases. This diet seems to form the basis for a better future, healthy life expectancy and longevity; therefore, after adaptation to local Polish conditions, it could be the basic component of MetS therapy.

### Study Strengths and Limitations

To the best of our knowledge, this was the first such study among Polish adults with metabolic disorders that included multiple factors simultaneously. We assessed the plasma levels of selected carotenoids and polyphenols in association with MetS severity, MetS components, inflammatory markers, and anthropometrics indices and related them to socio-demographic and lifestyle factors. However, the findings of this study should be considered in light of some limitations. First, the design of this epidemiological study only allows for the determination of the association between the variables, not their causal relationship. Although multivariate-adjusted logistic regression (multivariate linear regression analysis) analysis was performed, all additional confounding factors may not have been accounted for. Another limitation of this study may be the use of the FFQ to assess nutrition. Although it is the most commonly used tool in epidemiological studies, it is well known that it has the potential to either overestimate or underestimate dietary intake. Finally, the plasma concentration of the carotenoids and polyphenols demonstrated to associate with MetS and its components should also be considered in relation to their intake and other factors affecting their bioavailability, including diet composition, intestinal microbiota, health status, and individual genetic conditions.

It should be noted that some results reported in our study did not reach statistical significance. However, despite these limitations, the findings provide valuable initial insights and associations that add to the existing knowledge on the impact of bioactive compounds, including carotenoids and polyphenols, on the incidence of chronic diseases (e.g., CVD and diabetes). It also indicates the need for early nutritional interventions aimed at increasing the supply of these health-important antioxidants by increasing the supply of vegetables and fruits, products enriched with them, or dietary supplements.

## 5. Conclusions

A low concentration of carotenoids and polyphenols in the plasma of adult Poles with MetS was associated with an improper diet with a low consumption of plant products, including vegetables and fruit. Promoting and adapting a diet that includes elements of the Mediterranean in local conditions in MetS patients, and thus also in patients at a high risk of cardiovascular diseases, is the most important lifestyle modification strategy in the therapeutic management aimed at limiting the progression of metabolic syndrome. Health professionals should promote this type of diet as part of patient education in our specific environmental and cultural settings. However, it is still unclear whether plasma antioxidant levels are causally related to metabolic syndrome or are the result of the pathology of the syndrome, requiring further investigation.

## Figures and Tables

**Table 1:** Characteristics of the study group.

Variable	Total Group (n = 275)
Sex (%):	
men	42.5 (117)
women	57.5 (158)
Age (years):	53.9 ± 12.5
Education (%):	
primary and vocational	23.6 (65)
secondary	43.4 (119)
university	33.1 (91)
Physical activity (%):	
low	74.9 (206)
moderate	22.2 (61)
high	2.9 (8)
Smoking status (%):	
current smokers	25.1 (69)
former smokers	32.7 (90)
never smoked	42.2 (116)
AntiOxi Index (%)	42.9 ± 13.3
Anthropometrics	
BMI (kg/m^2^):	30.8 ± 7.0
men	30.7 ± 5.8
women	30.8 ± 7.8
WC (cm):	
men	110.6 ± 15.7
women	100.1 ± 16.9
WHtR:	0.6 ± 0.1
Fat mass (%):	
men	27.1 ± 8.1
women	38.1 ± 9.1
Biochemical markers and blood pressure	
Blood pressure (mmHg):	
systolic	135.7 ± 17.9
diastolic	81.1 ± 12.0
Cholesterol (mmol/L):	5.0 ± 0.9
Triglicerides (mmol/L):	2.0 ± 0.7
HDL cholesterol (mmol/L):	
men	1.0 ± 0.3
women	1.2 ± 0.3
LDL cholesterol (mmol/L):	2.7 ± 0.8
Fasting glucose (mmol/L):	7.2 ± 3.3
CRP (mg/L)	
men	3.3 ± 5.3
women	2.3 ± 4.5
Number of MetS-components:	
3	54.2 (149)
4	25.5 (70)
5	20.4 (56)
MetS severity:	
men	0.5 ± 1.2
women	0.8 ± 1.2

BMI—body mass index; CRP—C-reactive protein; HDL—high-density lipoprotein; LDL—low-density lipoprotein; MetS—metabolic syndrome; WC—waist circumference; WHtR—waist to hip ratio.

**Table 2:** Plasma carotenoids (µmol/L) by the number of MetS components among adults.

Plasma Carotenoids (µmol/L)	Total Group (n = 275)	MetS Components			*p*-Value *
		3 (n = 149)	4 (n = 70)	5 (n = 56)	
	Mean (95% CI) Min–Max				
Lutein	0.50 (0.46–0.54) 0.03–1.51	0.55 (0.49–0.60) ^a^ 0.07–1.51	0.51 (0.43–0.59) ^a^ 0.03–1.38	0.34 (0.28–0.40) ^b^ 0.06–0.89	≤0.001
Zeaxanthin	0.13 (0.12–0.14) 0.01–0.34	0.14 (0.13–0.16) ^a^ 0.01–0.34	0.13 (0.11–0.15) ^a^ 0.02–0.33	0.11 (0.09–0.13) ^b^ 0.02–0.31	0.008
L + Z	0.63 (0.59–0.67) 0.05–1.75	0.69 (0.63–0.75) ^a^ 0.14–1.75	0.64 (0.29–0.40) ^a^ 0.05–1.44	0.45 (0.39–0.51) ^b^ 0.08–0.96	≤0.001
Lycopene	0.30 (0.28–0.32) 0.02–0.75	0.29 (0.27–0.32) 0.02–0.72	0.30 (0.26–0.35) 0.03–0.75	0.29 (0.24–0.34) 0.08–0.71	0.844
β-carotene	0.30 (0.28–0.32) 0.05–0.74	0.29 (0.25–0.32) 0.05–0.74	0.29 (0.25–0.34) 0.08–0.72	0.34 (0.29–0.39) 0.08–0.73	0.143
Total carotenoids	1.22 (1.17–1.27) 0.38–2.58	1.27 (1.20–1.34) ^a^ 0.40–2.36	1.23 (1.12–1.35) ^a^ 0.38–2.58	1.08 (0.99–1.18) ^b^ 0.48–1.89	0.039

MetS components—number of metabolic syndrome criteria; * Kruskal–Wallis test; CI—confidence interval; L + Z—lutein + zeaxanthin; different letters indicate that the samples are significantly different at *p* < 0.05.

**Table 3:** Plasma polyphenols (nmol/L) by the number of MetS components among adults.

Plasma Polyphenols (nmol/L)	Total Group (n = 275)	MetS Components			*p*-Value *
		3 (n = 149)	4 (n = 70)	5 (n = 56)	
		Mean (95% CI) Min–Max			
Gallic acid	16.3 (15.3–17.3) 0.00–35.4	15.7 (14.4–17.0) 0.00–35.2	16.5 (14.4–18.6) 0.00–35.4	17.6 (15.3–19.8) 7.70–34.7	0.439
Chlorogenic acid	8.20 (7.70–8.60) 0.00–17.6	8.20 (7.60–8.80) 0.00–17.6	08.50 (7.50–9.60) 0.00–17.3	7.70 (6.70–8.60) 0.00–16.4	0.607
Caffeic acid	11.8 (11.2–12.4) 0.00–23.4	11.3 (10.5–12.1) 0.00–23.4	12.9 (11.5–14.2) 0.00–23.3	11.9 (10.5–13.0) 0.00–22.6	0.084
p-Coumaric acid	19.4 (17.6–21.2) 0.00–56.9	19.1 (16.7–21.6) 0.00–56.4	21.1 (17.1–25.1) 0.00–56.9	17.9 (14.3–21.5) 0.00–53.0	0.688
Ferulic acid	5.81 (5.20–6.32) 0.00–18.1	6.11 (5.41–6.93) 0.00–18.1	5.11 (4.10–6.22) 0.00–16.7	5.54 (4.22–6.80) 0.00–16.4	0.240
Phenolic acids	61.4 (58.9–63.9) 90.2–120.6	60.4 (57.0–63.9) 89.8–110.2	64.1 (58.5–69.7) 22.0–120.6	60.6 (55.5–65.8) 31.7–115.5	0.436
Kaempferol	3.5 (3.2–3.8) 0.00–0.93	3.7 (3.3–4.0) 0.00–9.3	3.3 (2.8–3.9) 0.00–8.5	3.3 (2.7–3.9) 0.00–8.2	0.389
Quercetin	14.4 (12.7–16.1) 0.00–48.6	14.9 (12.7–17.3) 0.00–45.7	12.2 (9.2–15.4) 0.00–44.6	15.6 (11.4–19.9) 0.00–48.6	0.338
Flavonol derivatives	17.9 (16.3–19.5) 0.00–57.7	18.6 (16.4–20.8) 0.00–54.3	15.6 (12.5–18.7) 0.00–52.4	18.9 (14.8–23.0) 0.00–57.0	0.323
Total polyphenols	79.3 (76.1–82.5) 12.7–162.5	79.0 (74.4–83.6) 12.7–162.5	79.7 (73.6–85.8) 22.1–137.9	79.5 (72.8–86.3) 33.8–148.6	0.732

MetS components—number of metabolic syndrome criteria; * Kruskal–Wallis test; CI—confidence interval.

**Table 4:** AntiOxi Index by quartiles and selected variables (expressed by mean ± SD and median).

Variables	AntiOxi Index				*p*-Value *
	Q1 (n = 68)	Q2 (n = 69)	Q3 (n = 70)	Q4 (n = 68)	
AntiOxi Index [%]	25.9 ± 6.5 27.0	38.9 ± 2.7 39.7	47.2 ± 2.6 46.8	59.1 ± 7.6 56.6	0.0001
Plasma lycopene (µmol/L)	0.28 ± 0.18 0.22 ^a^	0.27 ± 0.16 0.23 ^a^	0.28 ± 0.15 0.28 ^b^	0.35 ± 0.17 0.35 ^c^	0.0065
Total plasma carotenoids (µmol/L)	1.21 ± 0.42 1.17	1.13 ± 0.47 1.07	1.25 ± 0.48 1.21	1.30 ± 0.38 1.25	0.1008
Total plasma polyphenols (nmol/L)	79.7 ± 28.0 77.5	78.7 ± 24.6 74.9	77.5 ± 27.5 75.9	81.3 ± 27.9 83.6	0.7091
MetS severity	0.51 ± 0.98	0.70 ± 1.22	0.61 ± 1.37	0.84 ± 1.33	0.4285

* ANOVA (for parametric distributions) or ANOVA Kruskal–Wallis tests (for nonparametric distributions); different letters indicate that the samples are significantly different at *p* < 0.05.

**Table 5:** Plasma carotenoids and MetS severity, MetS components and CRP—results of multivariate linear regression analysis.

Plasma Carotenoids (µmol/L)	Multivariate Models β (95% CI) R^2^									
	MetS Severity	Fasting Glucose (mmol/L)	HDL (mmol/L)	LDL (mmol/L)	TG (mmol/L)	Cholesterol (mmol/L)	WC (cm)	SBP (mmHg)	DBP (mmHg)	CRP (mg/L)
Lutein	−0.105 (−0.225–0.015) R^2^ = 0.08 ***	−0.126 (−0.249–−0.003) * R^2^ = 0.04 *	0.088 (−0.028–0.204) R^2^ = 0.14 ***	0.003 (−0.121–0.127) R^2^ = 0.02	−0.011 (−0.134–0.113) R^2^ = 0.03	−0.028 (−0.152–0.097) R^2^ = 0.01	−0.079 (−0.152–−0.007) R^2^ = 0.66 ***	−0.089 (−0.214–0.036) R^2^ = 0.01	0.061 (−0.226–0.014) R^2^ = 0.08	−0.083 (−0.206–0.040) R^2^ = 0.04 *
Zeaxanthin	0.002 (−0.119–0.123) R^2^ = 0.07 **	−0.044 (−0.168–0.080) R^2^ = 0.02	0.157 (0.042–0.272) ** R^2^ = 0.16 ***	0.081 (−0.043–0.205) R^2^ = 0.03	0.047 (−0.077–0.171) R^2^ = 0.03	0.133 (0.009–0.257) * R^2^ = 0.02	−0.053 (−0.126–0.020) R^2^ = 0.66 ***	−0.068 (−0.193–0.057) R^2^ = 0.01	−0.076 (−0.197–0.045) R^2^ = 0.07 ***	−0.153 (−0.276–0.031) * R^2^ = 0.05 **
L + Z	−0.102 (−0.033–0.204) R^2^ = 0.08 ***	−0.134 (−0.258–−0.010) * R^2^ = 0.04 *	0.125 (0.008–0.241) * R^2^ = 0.14 ***	0.023 (−0.102–0.148) R^2^ = 0.02	0.001 (−0.124–0.126) R^2^ = 0.03	0.006 (−0.120–0.132) R^2^ = 0.01	−0.091 (−0.164–−0.017) R^2^ = 0.67 ***	−0.104 (−0.230–0.022) R^2^ = 0.01	−0.122 (−0.243–−0.001) R^2^ = 0.07 ***	−0.119 (−0.243–0.005) R^2^ = 0.03
Lycopene	0.086 (−0.033–0.204) R^2^ = 0.08 ***	−0.048 (−0.171–0.074) R^2^ = 0.03	0.031 (−0.068–0.158) R^2^ = 0.14 ***	0.066 (−0.056–0.188) R^2^ = 0.03	0.166 (0.046–0.286) ** R^2^ = 0.06 **	0.150 (0.029–0.272) * R^2^ = 0.03 *	0.000 (−0.073–0.072) R^2^ = 0.66 ***	−0.068 (−0.191–0.055) R^2^ = 0.01	−0.042 (−0.161–0.078) R^2^ = 0.07 ***	0.025 (0.096–0.147) * R^2^ = 0.03
β-carotene	−0.115 (−0.234–0.003) R^2^ = 0.08 ***	−0.061 (−0.183–0.061) R^2^ = 0.02	0.046 (−0.068–0.161) R^2^ = 0.14 ***	0.009 (−0.113–0.131) R^2^ = 0.02	0.069 (−0.052–0.191) R^2^ = 0.05 *	0.016 (−0.107–0.140) R^2^ = 0.01	−0.019 (−0.096–0.058) R^2^ = 0.66 ***	0.031 (−0.092–0.155) R^2^ = 0.00	−0.031 (−0.150–0.088) R^2^ = 0.07 ***	−0.025 (−0.147–0.096) R^2^ = 0.03
Total	−0.056 (−0.176–0.064) R^2^ = 0.08 ***	−0.148 (−0.271–−0.025) * R^2^ = 0.04 *	0.128 (0.012–0.243) * R^2^ = 0.15 ***	0.047 (−0.077–0.171) R^2^ = 0.02	0.096 (−0.027–0.219) R^2^ = 0.04 *	0.070 (−0.055–0.195) R^2^ = 0.01	−0.070 (−0.143–0.003) R^2^ = 0.66 ***	−0.092 (−0.217–0.033) R^2^ = 0.01	−0.123 (−0.243–−0.003) R^2^ = 0.08 ***	−0.092 (−0.215–0.031) R^2^ = 0.04 *

Multivariate models adjusted for sex, age, fat mass (%), education, smoking status, AntiOxi Index, and physical activity. CI—confidence intervals; HDL—high-density lipoprotein; LDL—low-density lipoprotein; L + Z—lutein and zeaxanthin. MetS—metabolic syndrome; TG—triglycerides, SBP—systolic blood pressure; DBP—diastolic blood pressure; CRP—C-reactive protein. * *p* ≤ 0.05; ** *p* ≤ 0.01; *** *p* ≤ 0.001.

**Table 6:** Plasma polyphenols and MetS severity, MetS components and CRP—results of multivariate linear regression analysis.

Plasma Polyphenols (nmol/L)	Multivariate Models β (95% CI) R^2^									
	MetS Severity	Fasting Glucose (mmol/L)	HDL (mmol/L)	LDL (mmol/L)	TG (mmol/L)	Cholesterol (mmol/L)	WC (cm)	SBP (mmHg)	DBP (mmHg)	CRP (mg/L)
Chlorogenic acid	−0.034 (−0.151–0.083) R^2^ = 0.05 **	−0.065 (−0.184–0.053) R^2^ = 0.02	0.017 (−0.094–0.128) R^2^ = 0.14 ***	0.138 (0.020–0.256) * R^2^ = 0.03 *	0.002 (−0.115–0.120) R^2^ = 0.04 **	0.125 (0.005–0.245) R^2^ = 0.01	0.068 (−0.003–0.138) R^2^ = 0.66 ***	0.042 (−0.079–0.164) R^2^ = 0.00	0.093 (−0.024–0.210) R^2^ = 0.08 ***	−0.116 (−0.235–0.003) R^2^ = 0.04 *
p-Coumaric acid	0.036 (−0.083–0.155) R^2^ = 0.05 **	−0.140 (−0.260–−0.021) * R^2^ = 0.04 *	0.016 (−0.096–0.129) R^2^ = 0.15 ***	−0.021 (−0.143–0.100) R^2^ = 0.01	−0.056 (−0.175–0.063) R^2^ = 0.04 *	0.027 (−0.096–0.149) R^2^ = 0.01	0.052 (−0.021–0.124) R^2^ = 0.66 ***	0.055 (−0.069–0.179) R^2^ = 0.00	0.096 (−0.024–0.215) R^2^ = 0.08 ***	−0.022 (−0.144–0.101) R^2^ = 0.03
Kaempferol	−0.042 (−0.159–0.075) R^2^ = 0.07 **	−0.017 (−0.136–0.102) R^2^ = 0.02	0.100 (−0.010–0.210) R^2^ = 0.16 ***	0.098 (−0.020–0.217) R^2^ = 0.02	0.063 (−0.054–0.181) R^2^ = 0.05 *	0.127 (0.008–0.247) * R^2^ = 0.01 *	−0.008 (−0.081–0.064) R^2^ = 0.66 ***	−0.031 (−0.154–0.092) R^2^ = 0.00	−0.011 (−0.130–0.108) R^2^ = 0.07 **	−0.075 (−0.196–0.046) R^2^ = 0.04 *

Multivariate models adjusted for sex, age, BMI, education, smoking status, AntiOxi Index, and physical activity. BMI—body mass index; CI—confidence intervals; HDL—high-density lipoprotein; LDL—low-density lipoprotein; MetS—metabolic syndrome; TG—triglycerides; WC—waist circumference; SBP—systolic blood pressure; DBP—diastolic blood pressure; CRP—C-reactive protein. * *p* ≤ 0.05; ** *p* ≤ 0.01; *** *p* ≤ 0.001.

## Data Availability

The data presented in this study are available on request from the corresponding author.
